# The NADPH Oxidase Inhibitor, Mitoapocynin, Mitigates DFP-Induced Reactive Astrogliosis in a Rat Model of Organophosphate Neurotoxicity

**DOI:** 10.3390/antiox12122061

**Published:** 2023-11-30

**Authors:** Christina Meyer, Elizabeth Grego, Suraj S. Vasanthi, Nikhil S. Rao, Nyzil Massey, Claire Holtkamp, Joselyn Huss, Lucas Showman, Balaji Narasimhan, Thimmasettappa Thippeswamy

**Affiliations:** 1 Department of Biomedical Sciences, Iowa State University, Ames, IA 50010, USA; cnameyer@iastate.edu (C.M.); surajsv@iastate.edu (S.S.V.); nikhilr@iastate.edu (N.S.R.); nyzil@iastate.edu (N.M.); claireh2@iastate.edu (C.H.); jjhuss@iastate.edu (J.H.); 2 Department of Chemical and Biological Engineering, Nanovaccine Institute, Iowa State University, Ames, IA 50011, USA; egrego@iastate.edu (E.G.); nbalaji@iastate.edu (B.N.); 3 W.M. Keck Metabolomics Research Laboratory, Iowa State University, Ames, IA 50011, USA; lshowman@iastate.edu

**Keywords:** NADPH oxidase (NOX), GP91*^phox^*, oxidative stress, astrogliosis, DFP (diisopropyl fluorophosphate), nanoparticles, K_ir_4.1

## Abstract

NADPH oxidase (NOX) is a primary mediator of superoxides, which promote oxidative stress, neurodegeneration, and neuroinflammation after diisopropylfluorophosphate (DFP) intoxication. Although orally administered mitoapocynin (MPO, 10 mg/kg), a mitochondrial-targeted NOX inhibitor, reduced oxidative stress and proinflammatory cytokines in the periphery, its efficacy in the brain regions of DFP-exposed rats was limited. In this study, we encapsulated MPO in polyanhydride nanoparticles (NPs) based on 1,6-bis(p-carboxyphenoxy) hexane (CPH) and sebacic anhydride (SA) for enhanced drug delivery to the brain and compared with a high oral dose of MPO (30 mg/kg). NOX2 (GP91*^phox^*) regulation and microglial (IBA1) morphology were analyzed to determine the efficacy of MPO-NP vs. MPO-oral in an 8-day study in the rat DFP model. Compared to the control, DFP-exposed animals exhibited significant upregulation of NOX2 and a reduced length and number of microglial processes, indicative of reactive microglia. Neither MPO treatment attenuated the DFP effect. Neurodegeneration (FJB+NeuN) was significantly greater in DFP-exposed groups regardless of treatment. Interestingly, neuronal loss in DFP+MPO-treated animals was not significantly different from the control. MPO-oral rescued inhibitory neuronal loss in the CA1 region of the hippocampus. Notably, MPO-NP and MPO-oral significantly reduced astrogliosis (absolute GFAP counts) and reactive gliosis (C3+GFAP). An analysis of inwardly rectifying potassium channels (K_ir_4.1) in astroglia revealed a significant reduction in the brain regions of the DFP+VEH group, but MPO had no effect. Overall, both NP-encapsulated and orally administered MPO had similar effects. Our findings demonstrate that MPO effectively mitigates DFP-induced reactive astrogliosis in several key brain regions and protects neurons in CA1, which may have long-term beneficial effects on spontaneous seizures and behavioral comorbidities. Long-term telemetry and behavioral studies and a different dosing regimen of MPO are required to understand its therapeutic potential.

## 1. Introduction

The development of epilepsy, i.e., epileptogenesis, is a prevailing outcome of status epilepticus (SE) [1,2]. Epileptogenesis is a critical period of disease progression in which cellular and molecular changes generate hyperexcitability in the brain. Consequently, neuronal death and gliosis, which occurs in the short term, could lead to spontaneously recurring seizures with behavioral and cognitive impairments in the long term [2,3]. Oxidative stress is a key mechanism that drives neurodegeneration and neuroinflammation during epileptogenesis [4]. NADPH oxidase (NOX) catalyzes the production of excessive reactive oxygen species (ROS) post-SE that causes oxidative stress and triggers epileptic seizures [5,6,7]. Isoforms of NOX are found in many brain cell types, including neurons (NOX1-4), microglia (NOX1, 2, and 4), and astrocytes (NOX1, 2, and 4) [8]. NOX, therefore, is a potential anti-epileptogenic target to modify cellular excitability in the brain. In the current study, we investigated the effects of mitochondrial-targeted NOX inhibitor mitoapocynin (MPO) in an organophosphate (OP) rat model of neurotoxicity.

OPs, including diisopropylfluorophosphate (DFP), induce SE by irreversibly inhibiting acetylcholinesterase. OP nerve agents have been employed in chemical warfare and terrorist attacks due to their highly toxic and volatile properties [9,10]. Unfortunately, no current medical countermeasures (MCMs) effectively mitigate the long-term effects of OP-exposure such as seizures, neurodegeneration, or neuroinflammation [11,12]. While the FDA-approved MCMs atropine, 2-PAM, and midazolam (MDZ) reduce acute mortality in early intervention, survivors still experience drastic long-term consequences such as anxiety, memory and learning deficits, and seizures [13,14]. Administration of MDZ within 10 min of exposure can rescue neurodegeneration; however, the limited time window for treatment is unrealistic in a real-world event [15,16,17]. Moreover, the current MCMs do not reduce NOX activation and its maladaptive mechanistic outcomes.

MPO has been shown to reduce mitochondrial dysfunction and oxidative stress and attenuate locomotor deficits in mouse models of Parkinson’s disease [18,19,20]. The triphenylphosphonium (TPP) moiety of MPO facilitates mitochondrial uptake and mitigates mitochondrial dysfunction [21,22]. In a model of Alzheimer’s disease, MPO reduced the opening of mitochondrial permeability transition pores [23]. Additionally, we previously discovered a reduction in inflammatory and oxidative stress markers by MPO in the serum, but not the brain, 8 days post-DFP [24]. While MPO was reported as having excellent bioavailability in the substantia nigra and the striatum of mice, hippocampal and cortical concentrations were the lowest compared to the other brain regions [18,19]. Considering the bioavailability concerns of MPO’s parent drug, apocynin, MPO likely presents a similar challenge for drug distribution to the hippocampus, the brain region that is markedly affected in epilepsy [1,25,26]. Pharmaceutical interventions for neurological disorders must penetrate the blood–brain barrier (BBB) to be effective. The BBB plays a largely beneficial role in protecting the brain from toxins and pathogens, though it often impedes certain drugs’ distribution to target regions, thus requiring a higher dose, which can cause toxicity [27]. To overcome this, we investigated nanoparticles (NP)-mediated drug delivery as an alternative approach compared to an elevated dose of oral MPO. Polyanhydride NPs are ideal drug carriers because they are biodegradable, and their rate of encapsulated payload release can be tailored to the desired experimental design [28,29,30].

The extent of BBB penetration by MPO-encapsulated polyanhydride NPs has been investigated in vitro. It was discovered that MPO-NP is internalized by human brain epithelial cells, a critical component of the BBB [31]. MPO-NP has not been investigated in vivo. In this study, we tested the efficacy of MPO-encapsulated NPs based on a 20:80 copolymer of 1,6-bis(p-carboxyphenoxy) hexane (CPH) and sebacic anhydride (SA) in an 8-day rat model of DFP-induced epileptogenesis. DFP exposure or seizures disrupt the BBB’s selective permeability [32]. Therefore, we compared the serum and hippocampal concentrations of MPO-NP (4 mg, i.m., every 2 days) and non-encapsulated MPO-oral (30 mg/kg, daily) dosing after exposing the rats to DFP. We hypothesized that following DFP exposure, NOX inhibition by MPO-NP or MPO-oral would reduce the markers of neurodegeneration and reactive microgliosis and astrogliosis in the hippocampus, amygdala, and piriform cortex. 

## 2. Materials and Methods

### 2.1. Materials

#### 2.1.1. Nanoparticle Synthesis and Characterization

**Figure 1:**
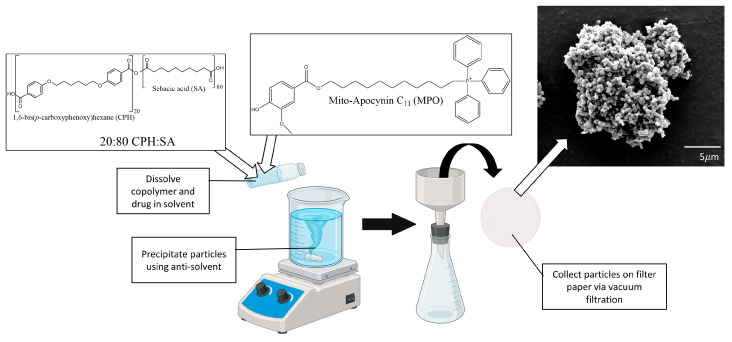
A schematic illustration of MPO-NP synthesis. CPH:SA and MPO were dissolved in methylene chloride, sonicated, and poured into a chilled pentane anti-solvent. MPO-NPs were collected by vacuum filtration and were scanned by an electron microscopy (SEM). Mean particle size was 415 ± 127 nm.

Sebacic acid (SA) was purchased from Sigma Aldrich, St. Louis, MO, USA. The synthesis of 1,6-bis(p-carboxyphenoxy) hexane (CPH) diacid was performed as previously described [29]. Melt polycondensation was used to synthesize 20:80 CPH:SA, as illustrated in Figure 1 [29,33]. Polymer purity, copolymer composition, and molecular weight were evaluated with 1H nuclear magnetic resonance spectroscopy (VXR 300 MHz, Varian, Palo Alto, CA, USA). 

A double oil emulsion method was used to synthesize polyanhydride NPs as previously described [34]. Desired copolymer and MPO were dissolved in methylene chloride and sonicated at 40 Hz for 30 s. Following sonication, the solution was poured into chilled pentane (−4 °C) at a solvent to non-solvent ratio of 1:250. NPs were immediately collected by vacuum filtration.

Particle size and morphology were evaluated by scanning electron microscopy (FEI Quanta, FEI, Hillsboro, OR, USA). Scanning electron microscopy samples were prepared by covering aluminum stubs with tape dusted with dry NPs. Particles were coated with a 5 nm layer of iridium using a Cressington 208 HR sputter coater (Watford, England, UK). An FEI Quanta 250 (FEI, Hillsboro, OR) instrument was used to collect particle images following coating. Particle sizing was performed with measuring tools available in Fiji. 

#### 2.1.2. MPO Loading and Release Kinetics

MPO loading was determined by suspending ~5 mg of particles in 1 mL of 40 mM sodium hydroxide for approximately 4 days with daily sample collection and washes to accelerate polymer degradation and drug release. Total drug released was quantified by an ultraviolet high-performance liquid chromatography (UV-HPLC) 1200 Series system (Agilent Technologies, Santa Clara, CA, USA). Samples were separated with a Phenomenex Kinetex 2.6 μm C18 100 Å 100 × 4.6 mM column with MPO analyzed at a wavelength of 262 nm. The sample flow rate was 1.5 mL/min with an initial mobile phase of 10:90 (%*v*/*v*) acetonitrile 0.1% trifluoroacetic acid (TFA)/water 0.1% TFA with a gradient ramping to 100 (%*v*/*v*) acetonitrile + 0.1% TFA for 7 min. As an isocratic step, the flow remained at 100 (%*v*/*v*) acetonitrile + 0.1% TFA for 10 min until a gradient ramp down to initial mobile phase conditions over 5 min. 

### 2.2. Animals

The Iowa State University (ISU) Institutional Care and Use Committee authorized animal care and procedures (IACUC-21-109) that complied with the NIH ARRIVE guidelines, including the sampling for pharmacokinetics, dosing regimens, DFP exposure, and euthanasia. Sprague Dawley rats, 7–8 weeks old, were purchased from Charles River Laboratories, Massachusetts, US. The rats were rested for 3 days after arrival to acclimate at the Laboratory of Animal Resources (ISU). All animals were housed individually in the same room with a 12 h light/dark cycle and ad libitum food and water. 

### 2.3. DFP Challenge, SE Monitoring, and Dosing Regimens

**Figure 2:**
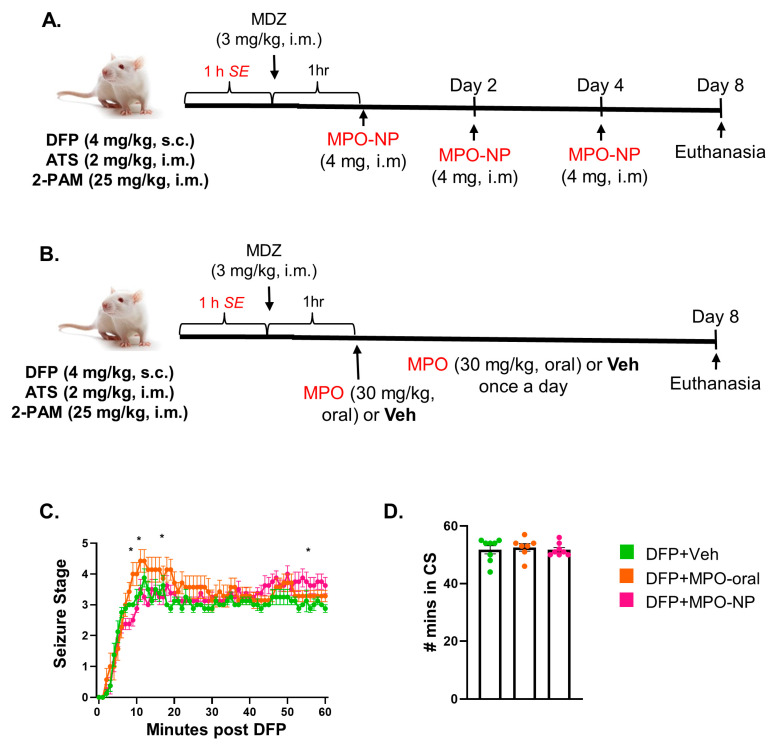
An experimental timeline of the dosing regimen and SE severity following DFP challenge. (**A**) MPO-NP animals were given three doses (4 mg, i.m.) every other day, beginning 2 h after DFP exposure i.e., 1 h post-MDZ. (**B**) DFP animals were treated with MPO (30 mg/kg, oral) or Vehicle (2% ethanol in dH_2_O, oral) received a daily dose until euthanized on day 8. (**C**) The seizure stage progression after DFP administration. Repeated measures two-way ANOVA. (**D**) The number of minutes spent in convulsive seizure (CS) in the 60 min following DFP. One-way ANOVA. n = 8, data are represented as mean ± SEM, * *p* < 0.05.

A timeline of DFP administration and treatment regimens are summarized in Figure 2A. DFP was purchased from Sigma-Aldrich (97.8% pure) and prepared in cold PBS. Control, DFP+Veh, and DFP+MPO were a mixed-sex cohort whereas DFP+MPO-NP animals were males (*n* = 8 per group). A total of 24 rats were challenged with DFP (4 mg/kg, s.c.) to induce status epilepticus (SE). To reduce acute mortality and peripheral effects, 25 mg/kg of 2-PAM (Sigma) and 2 mg/kg of atropine sulfate (ATS, Tokyo Chemical Industry) were given intramuscularly immediately after the DFP injection. As in previous studies, SE was monitored for an hour and scored by two experimenters using a modified Racine scale described in previous publications [24,35,36]. The duration of convulsive seizures (stages 3–5) between DFP and MDZ injections is an indicator of SE severity. The progression of seizures and the amount of time spent in SE greatly impacts the extent of brain pathology, reflected by the state of neurodegeneration, neuroinflammation, and the development of spontaneous recurrent seizures [37]. Therefore, animals were matched by minutes spent in convulsive seizures, i.e., SE severity, and then randomized into groups (n = 8 per group). Figure 2C,D demonstrates the seizure severity and progression post-DFP challenge. MDZ (3 mg/kg, i.m.) was purchased at the ISU Lloyd Veterinary Medical Center and administered to cease SE. Shortly after, 5% dextrose in normal saline (1 mL) was given subcutaneously. Animals rested for an hour before Vehicle, MPO C11 (American Biochemicals, TX), or MPO-NP treatment. Similar to our previous MPO study, animals in the DFP+Veh and DFP+MPO-oral received a daily dose of 2% ethanol in dH_2_O (oral) and MPO (30 mg/kg), respectively [24]. The dosing regimen for MPO-NP was chosen based on preliminary pharmacokinetics (Figure S1). The animals in the DFP+MPO-NP group were given 4 mg (19.5% MPO loaded, i.m.) every other day for three doses for a week. Vetoquinol Nutri-Cal, moistened food pellets, and 5% dextrose in normal saline (1 mL, s.c.) were provided until weight gain. 

### 2.4. Euthanasia and Tissue Collection

The animals were euthanized with 100 mg/kg pentobarbital sodium (i.p.) purchased from the Lloyd Veterinary Medical Center, ISU. Perfusion was performed with cold PBS. Then, one half of the brain was collected fresh and flash-frozen in liquid nitrogen for LC-MS, while the other was fixed in 4% paraformaldehyde (PFA, Acros Organics) for 2 days. PFA-fixed tissues were transferred to 25% sucrose in PBS for 3 days, as described in our previous publication [38]. The tissues were incubated in gelatin (15% type A porcine gelatin, 7.5% sucrose, 0.1% sodium azide) for 2 h at 37 °C and transferred to 4 °C overnight. The following day, tissues were blocked by rapidly freezing in 2-methylbutane immersed in liquid nitrogen. The tissue blocks were kept at −80 °C until cryo-sectioned (Thermo Fisher, cryostat, Waltham, MA, USA). The brains were sectioned rostral to caudal at a thickness of 16 µm and collected on chrome-alum gelatin-coated slides for immunohistochemistry (IHC). 

### 2.5. Mitoapocynin Extraction and Quantification

#### 2.5.1. Extraction of Mitoapocynin from Sera and Brain Tissue for LC-MS/MS

Serum (30–100 µL) and brain tissue (20–400 mg) samples were used to determine MPO concentrations. Samples were stored at −80 °C until extraction. High-quality LC-MS grade water, methanol, and acetonitrile (Fisher Scientific, Waltham, MA, USA) were used for preparing sample extracts and standards solutions. Prior to extraction, an internal standard was added to each sample, 10 µL of 2.5 µg/mL (2-hydroxy-3-phenoxy propyl) triphenylphosphonium bromide (Cat. No. S16161, Millipore Sigma, Burlington, MA, USA). Extractions began with the addition of 0.2 mL of methanol to all serum and tissue samples. Serum samples were then inverted, vortexed, and incubated on ice for 10 min before sonication for 5 min via a sonicating water bath (Model 2510, Branson Ultrasonics, Brookfield, CT, USA). Hippocampal samples were prepared for homogenization with the addition of two 2.4 mm metal grinding beads (Fisher Scientific, Waltham, MA, USA) to each sample. The tissue samples were then homogenized using a Bead Mill 24 Homogenizer (Thermo Fisher Scientific, Inc., Waltham, MA, USA). 0.8 mL of water was added to all samples before sonication for 5 min via a sonicating water bath. Samples were vortexed for 5 min prior to centrifugation at 16,300× *g* for 10 min. The extract supernatants were collected as the sample extracts. The extraction process was repeated on the remaining sample pellets with a volume of 1.0 mL of acetonitrile. The resulting extracts were combined and filtered with 0.2 µM centrifugal filters (Cat. No. UFC30LG25, Millipore Sigma, Burlington, MA, USA) before being subjected to LC-MS/MS analysis.

An MPO standard curve with a range of 0.01 to 2500 ng per sample was prepared as serial dilutions in 5:4:1 acetonitrile: water: methanol and combined with 75 µL of 0.9% NaCl to serve as a sample analog before being extracted and subjected to LC-MS/MS analysis in the same manner as the biological samples.

#### 2.5.2. Mitoapocynin LC-MS/MS Quantification

For LC-MS/MS MPO quantification, the liquid chromatography separations were performed with an Agilent Technologies 1290 Infinity II UHPLC instrument equipped with an Agilent ZORBEX Eclipse plus C18 analytical column (2.1 mM × 50 mm, 1.8 µM) that was coupled to a 6470 triple quadrupole mass spectrometer with an electrospray ionization (ESI) source (Agilent Technologies, Santa Clara, CA, USA). MPO sample extracts and standards were stored in the dark at 10 °C in the autosampler during LC-MS/MS analysis. A volume of 5 µL of each sample was injected into the LC system. The chromatography was carried out at 40 °C with a flow rate of 0.400 mL/min. All LC-MS/MS solvents used were LC-MS grade (Fisher Scientific, Waltham, MA, USA). Running solvents were A: water with 0.1% formic acid and B: acetonitrile with 0.1% formic acid. Initial solvent conditions were 15% B, which was held for 0.25 min before being decreased on a linear gradient to 100% B over 9.75 min, 100% B was held for 4 min before returning to 100% B over a 2 min linear gradient. A 4 min post run at 15% B was conducted after each LC-MS/MS acquisition.

The detector was operated using electrospray ionization in positive ionization mode. Nitrogen was used as the service gas for the ion source with a drying gas flow rate of 12 L/min at a temperature of 350 °C, a nebulizing pressure of 25 psi, and a sheath gas flow of 11 L/min at 375 °C. The capillary and nozzle voltages were 4000 and 0 volts, respectively. The mass spectrometer was operated in multiple reaction monitoring mode with two targeted transitions acquired: for MPO m/z 583.2 → 415.2 at a collision energy of 50 V and for the internal standard m/z 413.2 → 275.1 at a collision energy of 38 V. All transitions were observed with a 175 ms dwell time, while the fragmentor and cell accelerator wereheld at 135 and 5 volts, respectively. Data evaluation and peak quantitation were performed using Agilent MassHunter Qualitative Analysis (version 10.0) and Agilent MassHunter Quantitative Analysis (version 10.0) software (Agilent Technologies, Santa Clara, CA, USA). Target peaks were found at a 3.8 min retention time for internal standard and 5.7 min for MPO (Figure S2). MPO quantification was finally determined by relative abundance to the internal standard and MPO standard curve, before being made relative to the measured sample masses and volumes.

### 2.6. Immunohistochemistry and Fluoro-Jade B Staining (FJB)

The targets for IHC in this study include IBA1+GP91*^phox^* (microgliosis and oxidative stress markers), NeuN+FJB (neurodegeneration), and parvalbumin (an inhibitory neuronal marker), C3+GFAP (a reactive gliosis marker), and K_ir_4.1+GFAP (an astroglia functional marker). The slides for IHC staining were placed in a citric acid buffer (10 mM citric acid, 0.05% Tween 20, pH 6) for 25 min at 95 °C. Next, the slides were washed in PBS and placed in a Shandon rack. Blocking buffer was pipetted onto the slides to incubate for an hour before adding primary antibodies and left overnight at 4 °C. The following day, the slides were left at room temperature for 20 min, washed with PBS, and incubated with secondary antibodies for an hour. If biotinylated antibodies were used, the slides were washed with PBS (×3) and incubated with the conjugated antibody for an hour. Finally, the slides were washed with PBS and coverslip-mounted with DAPI-containing Vectashield Antifade mounting media. 

FJB staining was performed on the second day of NeuN IHC to quantify degenerating neurons. The slides were washed with PBS and dH_2_O, then incubated in 0.006% KMnO_4_ in dH_2_O for 5 min. Afterward, the slides were washed in dH_2_O and placed in 0.0003% FJB solution for 10 min in the dark. Lastly, they were air-dried, mounted with Surgipath Acrytol, and stored in the dark. 

### 2.7. Imaging, Cell Quantification, and Morphometric Analysis

Immuno-stained sections were imaged using a Leica DMi8 (Thermo Fisher) inverted fluorescent microscope. All images were captured at 20× with an 11 snapshot Z-stack and exported as Tiff files from the Leica Application Suite (LAS X). This study required a minimum of three brain sections per animal for cell quantification and morphometric analysis. For each section, we captured images of hippocampal regions CA1, CA3, and dentate gyrus (DG), as well as the amygdala (AMY) and piriform cortex (PC). Colocalization and absolute cell counts were quantified with the multi-point tool. For microglial morphometric analysis, we performed maximum contrast projection on R studio (EBImage package) and used a modified skeletonization protocol as previously described [39,40]. The values were then averaged and divided to calculate the mean number per microglia. We used a JACoP ImageJ plugin to determine the Pearson’s correlation coefficient to capture the overlap between K_ir_4.1 and GFAP [41]. 

### 2.8. Statistical Analyses

All experimenters were blinded to group conditions throughout the study by randomization and coding of the animals. Data were cross-verified by a second experimenter. The statistical program GraphPad Prism 9.0 (La Jolla, CA, USA) was used for data analysis and graphing. Normality was determined for each data set with the Shapiro–Wilk test, and depending on the results, a one-way ANOVA with a Tukey’s post hoc test for parametric or a Kruskal–Wallis with a Dunn’s post hoc test for non-parametric was performed. Sex interactions were tested using a two-way ANOVA and the results are presented in Supplementary Table S1. A two-way ANOVA mixed effects analysis determined the main group effects. Statistical significance was considered as *p* < 0.05.

## 3. Results

### 3.1. MPO-NP and MPO-Oral Concentrations in the Brain and Serum Post-DFP

**Figure 3:**
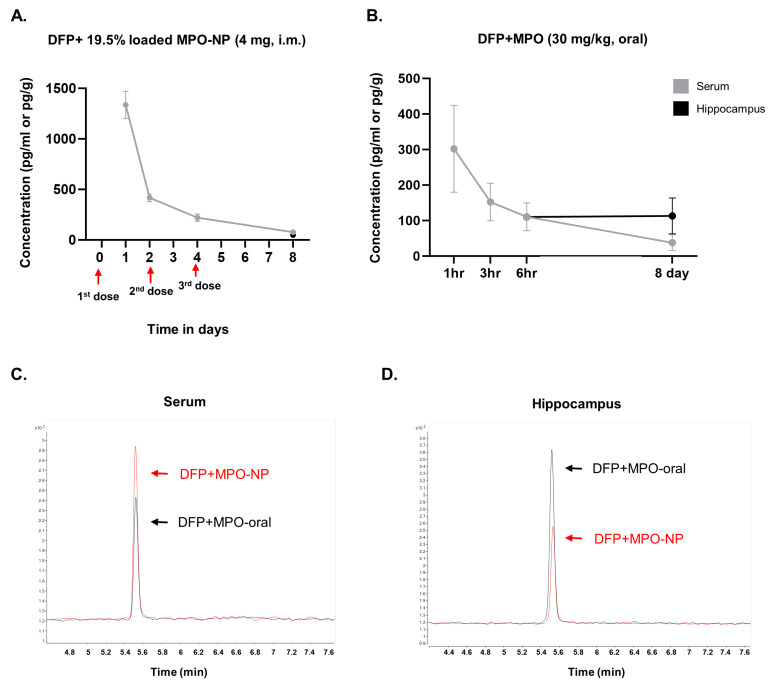
Serum and hippocampal MPO concentrations measured by LC/MS-MS. (**A**) MPO concentrations in DFP+MPO-NP (NPs 19.5% loaded) animals. Serum concentrations (pg/mL) decreased from 24 h to 48 h after the first dose, 48 h after the second dose, and on day 8 (72 h after the third dose). (**B**) MPO concentrations in DFP+MPO orally treated (30 mg/kg, daily) animals. Serum concentrations from 1 h to 6 h after the first dose. Hippocampal concentrations (pg/g) exceeded the serum on day 8 (24 h after the last dose). n = 4, data are represented as mean ± SEM. Chromatogram of MPO in the serum (**C**) and hippocampus (**D**) on day 8.

We performed LC-MS on serum and hippocampal brain tissue to evaluate the MPO concentration when administered orally or encapsulated in NPs after the DFP-challenge. The MPO payload within MPO-NP was 19.5 wt.%; each dose (4 mg, i.m.) contained 0.78 mg of MPO. MPO concentrations decreased from 24 to 48 h after the first dose and 48 h after the second dose. The concentrations at 72 h after the third and final MPO-NP dose were the lowest (Figure 3A). MPO serum concentrations decreased one hour after the first oral dose. MPO was detected in the brain 24 h after the final dose, suggesting brain permeability (Figure 3B). 

### 3.2. MPO-Oral Modifies Neuronal Loss Post-DFP

**Figure 4:**
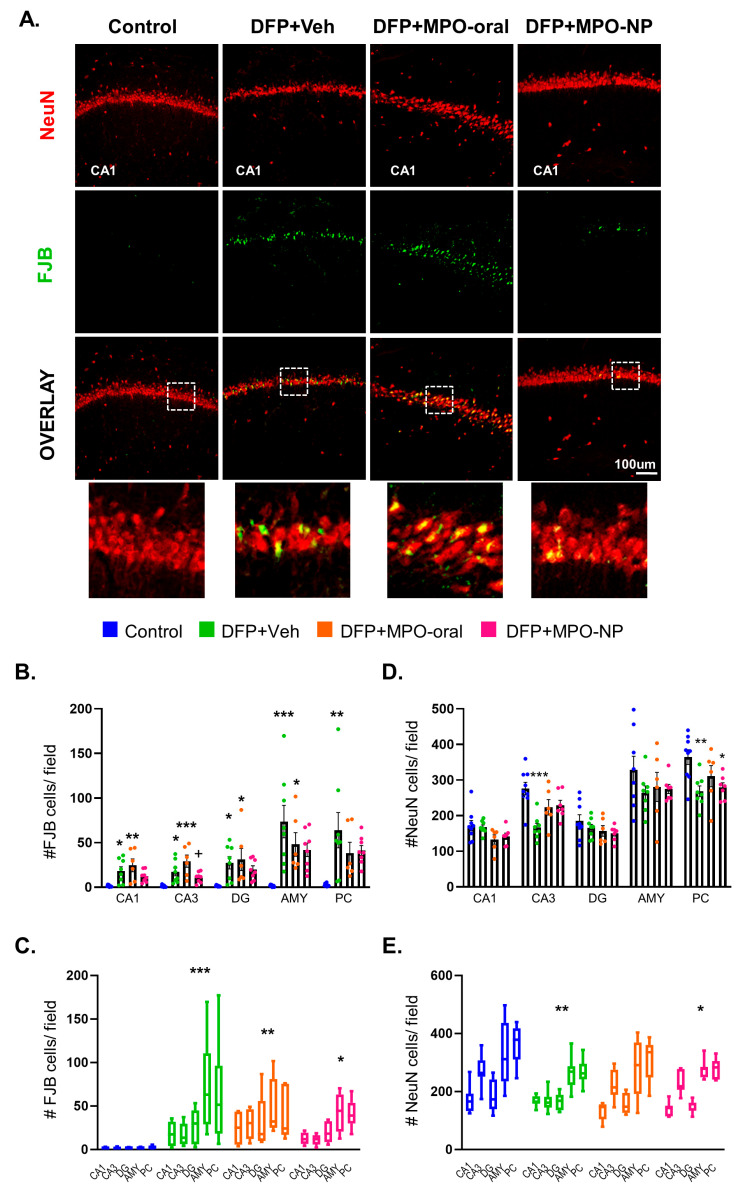
Neurodegeneration 8 days after DFP. (**A**) Representative IHC images of neurons (NeuN, red) in a degenerative state (FJB, green) in the CA1 region. Regional differences (**B**,**C**) and overall group effect on FJB-positive neurons. The colocalization was significantly greater in DFP+Veh animals in all regions quantified. FJB+NeuN was significantly reduced in the CA3 in DFP+MPO-NP. One-way ANOVA (**B**); two-way ANOVA mixed effects analysis (**C**). Regional differences (**D**,**E**) and overall group effect on absolute counts of NeuN-positive cells. Overall, there was a significant reduction in NeuN in DFP+Veh and DFP+MPO-NP, but not DFP+MPO-oral treated animals. One-way ANOVA (**D**); two-way ANOVA mixed effects analysis (**E**). n = 8, data are represented as mean ± SEM. * *p* < 0.05, ** *p* < 0.01, *** *p* < 0.001 compared to control. + *p* < 0.05 compared to DFP+MPO-oral.

To determine the extent of neurodegeneration, we analyzed the number of NeuN-FJB colocalization and absolute NeuN-positive cells. Animals in the DFP+Veh group showed a significant increase in FJB-NeuN colocalization in all brain regions quantified compared to the control. Overall, DFP animals treated with either MPO-oral or MPO-NP had no significant reduction in FJB-positive neurons. In the CA3, FJB in DFP+MPO-NP was significantly lower than DFP+MPO-oral (Figure 4B). However, there was a significant group effect of all DFP groups when compared to the control (Figure 4C). A sex interaction of FJB+NeuN was observed in the DG (Table S1).

The effect of DFP+Veh on absolute NeuN count was less pronounced, with a significant decrease observed in only CA3 and PC regions (Figure 4D). In the grouped regions, DFP+VEH and DFP+MPO-NP, but not DFP+MPO-oral, a significant decrease in NeuN was observed compared to the control (Figure 4E). There was a sex interaction of absolute NeuN in the DG and AMY (Table S1).

### 3.3. MPO-Oral Mitigates GABAergic Neuronal Loss in the CA1 Hippocampal Region Post-DFP

**Figure 5:**
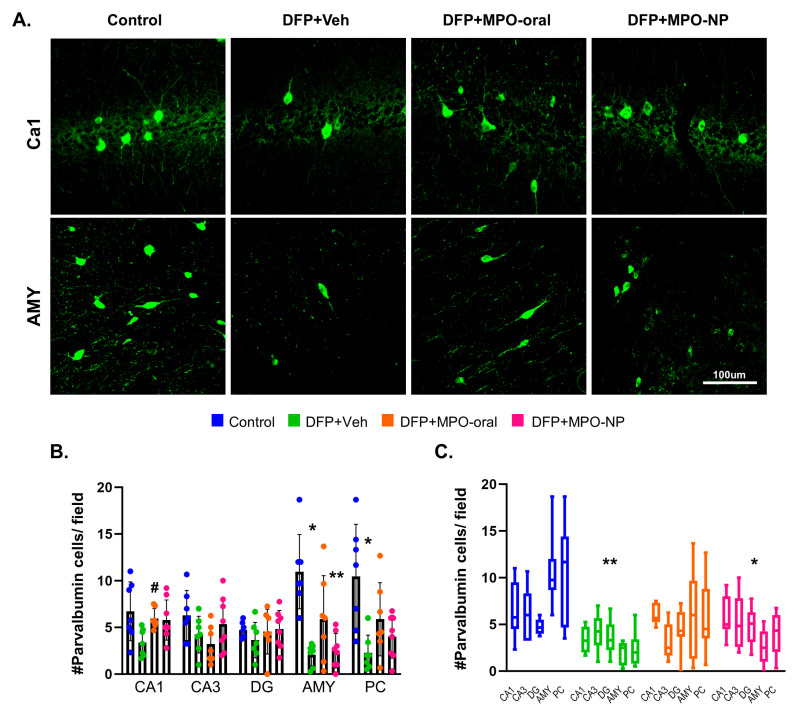
Parvalbumin (PV) interneurons 8 days after DFP. (**A**) Representative IHC images of parvalbumin-positive neurons in the CA1 and piriform cortex (PC). (**B**) Parvalbumin-positive neurons were significantly decreased in DFP+Veh animals in the amygdala (AMY) and PC. In the CA1 region, MPO-oral significantly attenuated DFP-induced PV+ve interneuronal loss. One-way ANOVA. (**C**) A two-way ANOVA mixed effects analysis revealed a significant reduction in parvalbumin-positive cells in DFP+Veh and DFP+MPO-NP compared to control. n = 8, data are represented as mean ± SEM. * *p* < 0.05, ** *p* < 0.01 compared to control. # *p* < 0.05 compared to DFP+Veh.

Inhibitory neuronal loss implies hyperexcitability [42,43]. Parvalbumin immunostaining revealed a significant loss of inhibitory neurons in the AMY and PC of vehicle animals challenged with DFP. MPO-oral treatment significantly mitigated parvalbumin loss in the CA1 (Figure 5B) but not in other regions. Overall, DFP+MPO-oral was not statistically different from the control, whereas there was a significant group effect of DFP+Veh and DFP+MPO-NP, but not DFP+MPO-oral on parvalbumin-expressing neurons (Figure 5C). No sex interactions were observed.

### 3.4. MPO-Oral and MPO-NP Treatment Did Not Affect NOX2 (GP91^phox^) Expression Post-DFP

**Figure 6:**
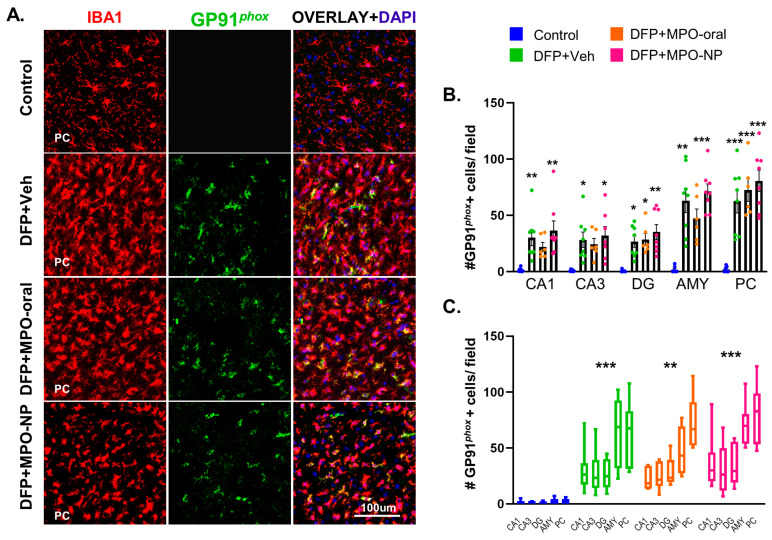
GP91*^phox^* expression 8 days after DFP. (**A**) Representative IHC images of microglia (IBA1, red) and NOX2 (GP91*^phox^*, green) in the piriform cortex (PC). (**B**) GP91*^phox^* expression in microglia was significantly increased in DFP+Veh and DFP+MPO-NP in the CA1, CA3, dentate gyrus (DG), amygdala (AMY), and PC. DFP+MPO-oral only saw a significant increase in DG and PC. Kruskal–Wallis test (CA1, DG, AMY), one-way ANOVA (CA3, PC). (**C**) There was a significant group effect of all DFP groups. Two-way ANOVA mixed effects analysis. n = 8, data are represented as mean ± SEM. * *p* < 0.05, ** *p* < 0.01, *** *p* < 0.001 compared to the control.

NOX2-colocalization with microglia in PC after PBS or DFP exposure is represented in Figure 6A. In each region quantified, including CA1, CA3, DG, AMY, and PC, GP91*^phox^* was significantly upregulated following DFP exposure (Figure 6B). In the CA1 and CA3, post-DFP treatment with MPO-oral was not significantly different from the control. However, as a main group effect, DFP+Veh, DFP+MPO-oral, and DFP+MPO-NP treatment groups showed a significant increase in GP91*^phox^* expression across regions (Figure 6B). MPO, either via oral or NP, had no effect. No sex interactions were observed.

### 3.5. DFP-Exposure Promoted Microglial Reactivity

**Figure 7:**
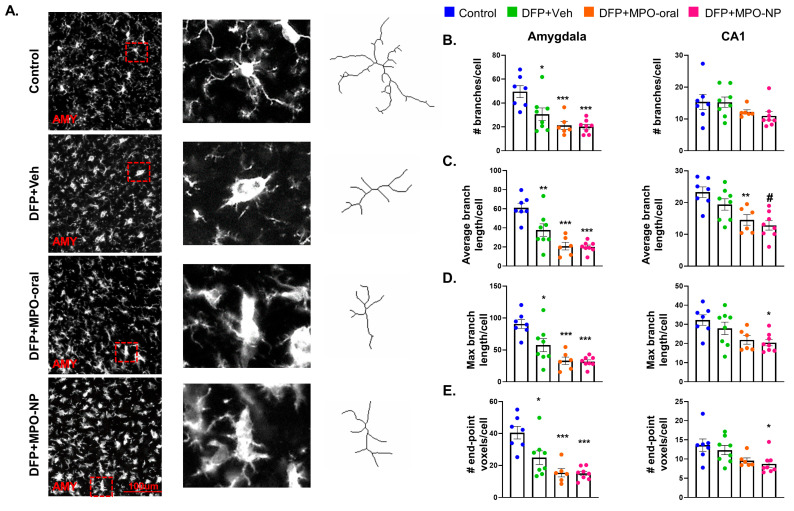
Microglia morphology 8 days after DFP. (**A**) Representative images of the morphometric analysis in the amygdala (AMY). (**B**–**E**) A comparison of the number of branches, average and maximum branch length, and the number of end-point voxels of microglia in the AMY and CA1 regions. Neither MPO-oral nor MPO-NP mitigated DFP-induced reactivity. One-way ANOVA. n = 8, data are represented as mean ± SEM. * *p* < 0.05, ** *p* < 0.01, *** *p* < 0.001 compared to control. # *p* < 0.05 compared to DFP+Veh.

We conducted a morphometric analysis of microglia in the AMY and CA1 to detect cellular reactivity. Figure 7A illustrates our method to quantify the microglial response to DFP exposure and the effects of MPO-oral and MPO-NP treatment. The number of branches (Figure 7B), average branch length (Figure 7C), maximum branch length (Figure 7), and the number of end-point voxels (Figure 7E) were analyzed. DFP, regardless of treatment, significantly reduced each parameter, demonstrating reactive-like cells in the AMY. There was no DFP effect in the CA1 region, despite the fact that the average branch length of the DFP+MPO-NP was significantly lower than DFP+Veh. The average branch length of the DFP+MPO-oral as well as the max branch length and end-point voxels in the DFP+MPO-NP group were significantly lower than the control in the CA1 (Figure 7C–E). No sex interactions were observed.

### 3.6. MPO-Oral and MPO-NP Attenuated Astrogliosis and Their Reactivity 8 Days Post-DFP

**Figure 8:**
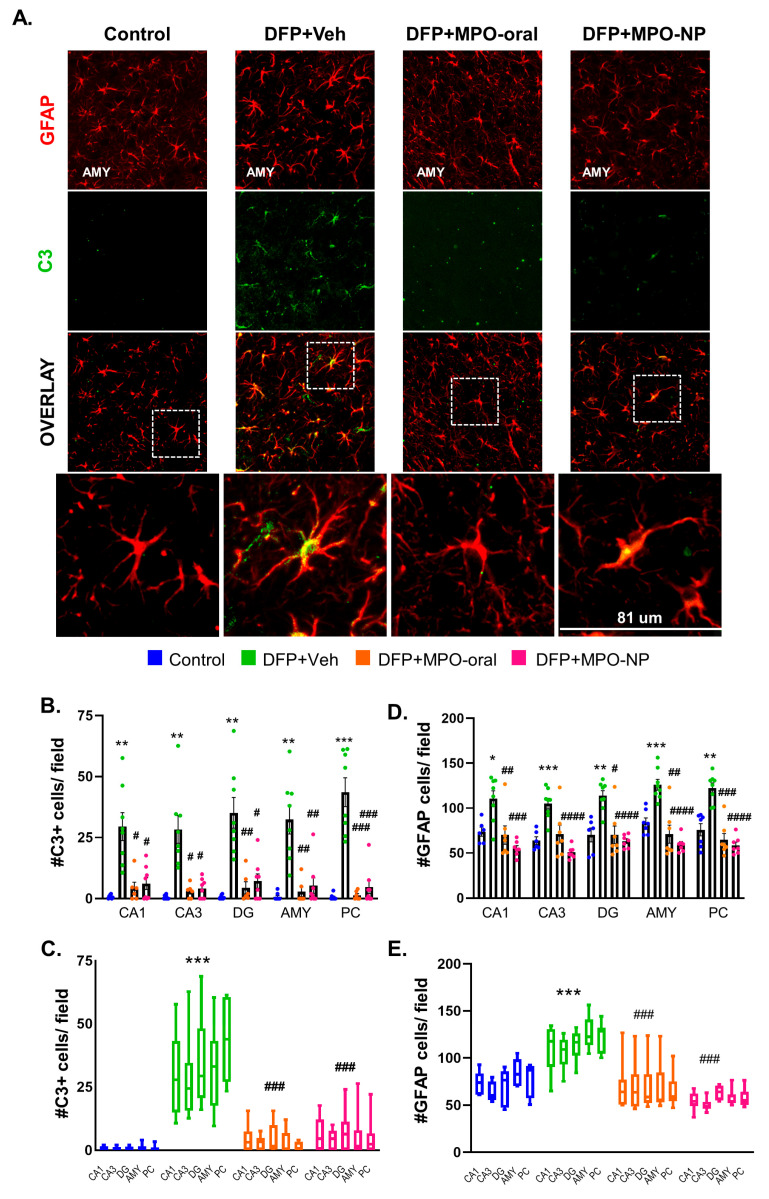
Astrogliosis 8 days after DFP. (**A**) Representative IHC images of complement 3- positive (C3, green) astroglia (GFAP, red) in the amygdala (AMY). (**B**,**C**) C3, GFAP colocalization was significantly decreased in DFP+MPO-oral and DFP+MPO-NP in the CA1, CA3, dentate gyrus (DG), AMY, and piriform cortex (PC). (**D**,**C**) Absolute GFAP counts were significantly reduced in DFP animals treated with MPO-oral or MPO-NP compared to the Vehicle. One-way ANOVA (**B**,**D**); two-way ANOVA mixed effects analysis (**C**,**E**). n = 8, data are represented as mean ± SEM. * *p* < 0.05, ** *p* < 0.01, *** *p* < 0.001. *** *p* < 0.001 compared to control. # *p* < 0.05, ## *p* < 0.01, ### *p* < 0.001. #### *p* < 0.0001 compared to DFP+Veh.

We quantified complement 3 (C3), GFAP superimposition, and an absolute GFAP count to measure astrocyte reactivity and astrogliosis. In response to DFP, we observed a significant upregulation of C3+GFAP in Veh-treated animals (Figure 8B,C). Treatment with MPO-oral and MPO-NP significantly mitigated C3 expression following DFP. The same pattern was observed in astrogliosis (increase in absolute numbers of GFAP+ cells) in the DFP+Veh. The number of GFAP-positive cells was significantly reduced in DFP+MPO-oral and DFP+MPO-NP compared to DFP+Veh (Figure 8D,E). No sex interactions were observed.

### 3.7. K_ir_4.1 Is Downregulated in Astrocytes after DFP Exposure

**Figure 9:**
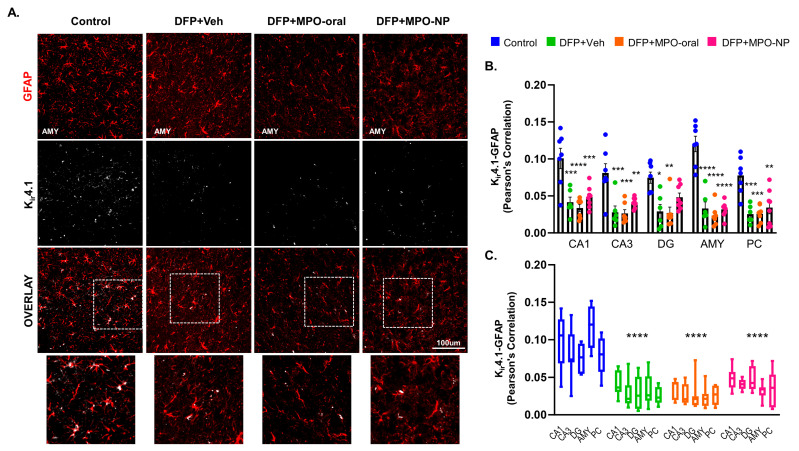
K_ir_4.1 8 days after DFP. (**A**) Representative IHC images of inward rectifying potassium channel 4.1 (K_ir_4.1, white) and astroglia (GFAP, red) in the amygdala (AMY). One-way ANOVA (**B**) and two-way ANOVA mixed effect analysis (**C**) revealed a significant loss of K_ir_4.1 in response to DFP, regardless of treatment group. n = 8, data are represented as mean ± SEM. * *p* < 0.05, ** *p* < 0.01, *** *p* < 0.001. **** *p* < 0.0001 compared to control.

Expression of K_ir_4.1 in astrocytes was determined by a correlation analysis of fluorescent overlap with GFAP (Figure 9A). K_ir_4.1 is a measure of astrocytes’ ability to regulate extracellular potassium. Following DFP challenge, K_ir_4.1 was significantly downregulated in all quantified regions. Other than DFP+MPO-NP Kir4.1 expression in the DG, neither MPO-oral nor MPO-NP treatment modified the DFP-induced effects. The same results were observed when the regions as a whole were analyzed (Figure 9B,C). No sex interactions were observed.

## 4. Discussion

Mitoapocynin (MPO) is a mitochondrial-targeted NOX inhibitor though the linkage of apocynin to TPP by an alkyl chain [19]. MPO effectively reduced oxidative stress and mitochondrial dysfunction in Parkinson’s disease, Alzheimer’s disease, kainic acid excitotoxicity, and organic dust exposure models [18,19,44,45]. In the rat model of DFP exposure, MPO reduced ROS and inflammatory markers in the periphery but not in the brain, which is vulnerable to oxidative injury due to its high oxygen consumption and abundance of polyunsaturated fatty acids [24,46]. Oxidative stress in the brain promotes neurodegeneration and neuroinflammation and is, therefore, a driving mechanism of epileptogenesis following OP exposure [47]. It is imperative that therapeutics aimed at mitigating epileptogenesis adequately penetrate the brain to yield anticipated outcomes. We encapsulated MPO in 20:80 CPH:SA NPs anticipating enhancing its bioavailability. Following the DFP-challenge, animals were given either MPO-NP (4 mg containing 19.5% MPO i.e., 0.78 mg, i.m.), MPO (30 mg/kg oral), or Vehicle (oral). MPO efficacy was investigated by quantifying neuronal loss and glial outcomes indicative of oxidative stress and reactivity. 

Spontaneous recurrent seizures typically begin within 2 weeks of OP exposure [48,49]; hence, an 8-day OP study is the optimal time to capture epileptogenic pathology. DFP and other OPs are known to cause excitatory and inhibitory neuronal loss. The significant increase in FJB-stained neurons observed in the current study suggests the effects of OPs in the short term. Neither MPO-oral nor MPO-NP altered the neurodegenerative outcome of DFP. While FJB+NeuN staining captures actively dying neurons, a measurement of absolute neuronal count (NeuN positive cells) reveals the neurons’ population in the brain regions of interest. Here, we discovered significant neuronal loss in the CA3 and PC regions. Nevertheless, when considering brain regions as a whole, there was a significant loss in the DFP+Veh and DFP+MPO-NP groups. MPO-oral mildly rescued neuronal loss in the brain as a whole, such that there was no significant difference in the neuronal count between DFP-MPO-oral and naive controls. 

The parvalbumin (PV) subpopulation of inhibitory neurons regulates excitatory pathways in the hippocampus and extrahippocampal regions in epilepsy [50]. In our 8-day DFP model, there was a significant loss of PV neurons in the hippocampus, AMY, and PC of DFP+Veh animals. Treatment with MPO-oral post-DFP significantly mitigated PV loss in the CA1, a selectively vulnerable region to oxidative stress [51]. Similar to NeuN, there was no overall difference in the presence of PV in the brains of MPO-treated animals. PV interneurons are implicated in anxiety and depression disorders; therefore, behavioral tests are needed to determine if MPO can alleviate behavioral comorbidities that follow OP poisoning [52]. In a model of post-traumatic stress disorder, apocynin improved cognitive deficits and PV loss by inhibiting NOX2 [53]. PV neurodegeneration is mediated by NOX-catalyzed ROS [43,54].

NOX2 also promotes microglial reactivity [55]. Microglia are essential in the brain as they are the resident immune cells and the first responders to insult. During a state of injury, microglia proliferate and release inflammatory cytokines and ROS in excess [56,57]. We have previously shown a marked upregulation of the NOX2 subunit GP91*^phox^* in microglia after exposure to DFP, demonstrating that it is a robust marker of pathology [36,58]. In prior studies, diapocynin was shown to reduce increased NOX2 activity by DFP, whereas MPO-oral did not [24,36]. In this study too, MPO-oral 30 mg/kg and MPO-NP did not impact GP91*^phox^* expression, which could be attributed to MPO’s affinity to the mitochondria compared to the cell membrane where GP91*^phox^* is located [59]. An additional indicator of microglial reactivity is altered cellular morphology, wherein cells exhibit retracted branch processes and enlarged soma [60,61,62]. In our study, DFP significantly reduced the number, length, and end-point voxels of branches in microglia one week after exposure regardless of treatment. The increase in amoeboid-shaped microglia supports the theory that cells were in a state of reactivity that promotes excitotoxicity in the brain. Microglial reactivity was more pronounced in the AMY than in the CA1 region. Interestingly, treatment with MPO-oral and MPO-NP reduced branching patterns in both regions. This finding was only significant by MPO-NP in the CA1, where the average and maximum branch length along with the number of end-point voxels were reduced. 

Astrocytes, like microglia, become reactive in response to brain injury. Complement 3 (C3) secretion is an important indicator of reactivity as an activator of microglia [63,64]. Additionally, C3 interacts with neurons by localizing to the synapses and marking them for pruning [65]. In the current study, C3 was significantly upregulated in DFP+VEH animals, signifying a reactive response of astrocytes to OP exposure. Notably, both MPO-NP and MPO-oral significantly attenuated this C3 expression. Astrogliosis was also mitigated by MPO-NP and MPO-oral treatment. The differential effects of MPO on astroglia and microglia could be due to the NOX isoforms expressed in the two cell types in response to the insult. Of the seven different forms of NOX, NOX1 and NOX4 reside in astroglia [8]. Remarkably, NOX4 is the only type that is localized to the mitochondria, which internalizes MPO [66]. Therefore, it is likely that MPO has a differential impact on microglia and astroglia. In a kainic acid model of excitotoxicity, MPO reduced total NOX4 expression in the striatum [44]. Together, this implies that MPO preferentially targets mitochondrial NOX4 in astrocytes instead of microglial NOX2.

Astrocytes are key players in epileptogenesis due to their role as regulators of neuronal excitability [67]. Inwardly rectifying potassium channels (K_ir_) on astrocytes govern excitability by regulating extracellular potassium ions [68,69]. K_ir_4.1 reduction leads to an accumulation of extracellular potassium and impairs the clearance of glutamate, which reduces the seizure threshold [70,71]. Reduction in K_ir_4.1, genetic or otherwise, is a feature of many types of epilepsy, such as temporal lobe epilepsy [72,73]. In the current OP model, there was a significant decrease in K_ir_4.1 in response to DFP exposure. This finding establishes a novel commonality between OP-induced epilepsy and other models of epilepsy such as the pentylenetetrazole model [74]. Conversely, epilepsy due to DFP can be distinguished from the cases of epilepsy-autism comorbidity wherein K_ir_4.1 membrane expression is increased [75]. 

In summary, despite the enhanced MPO serum concentrations with MPO-NP, the effects of MPO-NP and MPO-oral were similar. The hippocampal concentrations of MPO in the animals receiving the NP formulation were lower than those of the orally treated group. However, the discrepancy could be due to the differing dosing regimen before blood collection. A detailed pharmacokinetic study of MPO-NP and MPO-oral is warranted with consideration of route of administration of NPs (nasal vs i.m.). Nevertheless, the impact of MPO 8 days post-DFP, whether encapsulated in NPs or oral, on astrogliosis and astrocyte reactivity may mitigate the progression of epileptogenesis or brain pathology in the long term by reducing the acute neuroinflammatory response. Interestingly, MPO-oral, but not MPO-NP, modified overall neuronal and PV inhibitory neuronal loss, wherein there was no difference between DFP+MPO-oral and control. An extended preclinical OP study evaluating spontaneous recurrent seizures and animal behavior will elucidate the long-term effects of astrogliosis mitigation and neuronal modification by MPO.

## Data Availability

The data is contained within the article and Supplementary Materials. The raw data presented in this study are also available upon request to the corresponding author: tswamy@iastate.edu.

## Supplementary Materials

The following supporting information can be downloaded at: https://www.mdpi.com/article/10.3390/antiox12122061/s1, Figure S1: Serum and hippocampus MPO concentrations; Table S1: Sex interaction statistics; Table S2: Antibodies used for immunohistochemistry; Figure S1: Preliminary kinetics of MPO-NP; Figure S2: Method validation of LCMS.
