# Supplementary material for: The NADPH Oxidase Inhibitor, Mitoapocynin, Mitigates DFP-Induced Reactive Astrogliosis in a Rat Model of Organophosphate Neurotoxicity
Source: Antioxidants (Basel). 2023 Nov 30;12(12):2061. doi: 10.3390/antiox12122061 (PMC10740988; doi:10.3390/antiox12122061)
Supplement: Supplementary file 1 [file antioxidants-12-02061-s001.zip › antioxidants-2719182-supplementary.pdf]

Table S1. Mean ±SEM values & statistical tests for sex-interaction.

Figure 3B	Control	DFP+ VEH	DFP + MPO-oral	Interaction effects P value	Statistical analysis
CA1	1.269 ± 0.313	18.24 ± 4.749	24.63 ± 7.17	0.2176	2-way ANOVA (Tukey's multiple comparison)
CA3	1.413± 0.4040	17.14± 4.399	29.30± 6.844	0.3146	2-way ANOVA (Tukey's multiple comparison)
DG	1.337± 0.2446	27.48± 6.971	31.17± 12.47	0.0154	2-way ANOVA (Tukey's multiple comparison)
AMY	1.569± 0.3224	73.63± 18.16	48.00± 13.30	0.2084	2-way ANOVA (Tukey's multiple comparison)
PC	2.561± 0.5447	63.98± 19.76	38.49± 11.93	0.3046	2-way ANOVA (Tukey's multiple comparison)
**Figure 3D**	**Control**	**DFP+ VEH**	**DFP + MPO-oral**	**Interaction effects P value**	**Statistical analysis**
CA1	172.0± 14.28	167.6± 6.341	133.1± 12.92	0.3438	2-way ANOVA (Tukey's multiple comparison)
CA3	276.0± 17.48	166.8± 11.73	223.5± 21.58	0.5437	2-way ANOVA (Tukey's multiple comparison)
DG	185.8± 16.82	165.6± 9.870	156.9± 14.77	0.0016	2-way ANOVA (Tukey's multiple comparison)
AMY	357.2± 45.07	264.2± 19.23	280.5± 40.81	0.0090	2-way ANOVA (Tukey's multiple comparison)
PC	364.6± 20.78	268.4± 15.16	311.0± 29.78	0.6318	2-way ANOVA (Tukey's multiple comparison)
**Figure 4B**	**Control**	**DFP+ VEH**	**DFP + MPO-oral**	**Interaction effects P value**	**Statistical analysis**
CA1	6.726± 1.188	3.381± 0.5512	5.952± 0.4017	0.3701	2-way ANOVA (Tukey's multiple comparison)
CA3	6.286± 1.012	4.238± 0.7401	3.202± 0.7537	0.1586	2-way ANOVA (Tukey's multiple comparison)
DG	4.702± 0.3201	3.643± 0.7161	4.548± 0.9023	0.0653	2-way ANOVA (Tukey's multiple comparison)
AMY	10.96± 1.500	2.098± 0.4483	5.893± 1.760	0.6707	2-way ANOVA (Tukey's multiple comparison)
PC	10.46± 2.108	2.279± 0.7183	5.876± 1.483	0.2004	2-way ANOVA (Tukey's multiple comparison)
**Figure 5B**	**Control**	**DFP+ VEH**	**DFP + MPO-oral**	**Interaction effects P value**	**Statistical analysis**
CA1	1.424± 0.6641	30.16± 7.040	22.09± 3.969	0.6868	2-way ANOVA (Tukey's multiple comparison)
CA3	0.8694± 0.3904	28.36± 6.836	24.48± 4.935	0.7271	2-way ANOVA (Tukey's multiple comparison)
DG	0.5643± 0.3796	26.66± 4.915	28.60± 5.300	0.6095	2-way ANOVA (Tukey's multiple comparison)
AMY	1.798± 1.045	63.04± 10.61	47.17± 8.564	0.2019	2-way ANOVA (Tukey's multiple comparison)
PC	2.000± 0.9350	62.58± 10.36	72.43± 9.984	0.6973	2-way ANOVA (Tukey's multiple comparison)

Figure 6 AMY	Control	DFP+ VEH	DFP + MPO-oral	Interaction effects P value	Statistical analysis
# Branch/cell	49.56± 4.966	30.63± 5.273	21.30± 3.361	0.2309	2-way ANOVA (Tukey's multiple comparison)
Average branch/cell	61.15± 4.644	37.73± 6.509	20.90± 4.012	0.0523	2-way ANOVA (Tukey's multiple comparison)
Max branch length	90.56± 6.915	57.37± 10.09	33.00± 5.818	0.3545	2-way ANOVA (Tukey's multiple comparison)
# end point voxels	40.45± 3.915	24.85± 4.234	15.42± 2.579	0.5017	2-way ANOVA (Tukey's multiple comparison)

Figure 6 CA1	Control	DFP+ VEH	DFP + MPO-oral	Interaction effects P value	Statistical analysis
# Branch/cell	15.36± 2.348	15.27± 1.612	12.21± 0.6777	0.6659	2-way ANOVA (Tukey's multiple comparison)
Average branch/cell	23.28± 1.659	19.40± 1.786	14.52± 1.661	0.8540	2-way ANOVA (Tukey's multiple comparison)
Max branch length	32.25± 2.735	27.90± 3.250	21.87± 2.267	0.7849	2-way ANOVA (Tukey's multiple comparison)
# end point voxels	13.60± 1.607	12.30± 1.187	9.580± 0.6965	0.7141	2-way ANOVA (Tukey's multiple comparison)

Figure 7B	Control	DFP+ VEH	DFP + MPO-oral	Interaction effects P value	Statistical analysis
CA1	0.7813± 0.2611	29.50± 5.703	4.417± 2.365	0.2006	2-way ANOVA (Tukey's multiple comparison)
CA3	0.5417± 0.2813	28.33± 5.698	3.028± 1.150	0.4261	2-way ANOVA (Tukey's multiple comparison)
DG	0.6250± 0.2777	35.14± 6.307	4.472± 2.512	0.2099	2-way ANOVA (Tukey's multiple comparison)
AMY	0.7500± 0.5261	32.40± 5.949	2.833± 2.007	0.4943	2-way ANOVA (Tukey's multiple comparison)
PC	0.6042± 0.4106	43.62± 5.888	1.389± 0.7119	0.1935	2-way ANOVA (Tukey's multiple comparison)

Figure 7D	Control	DFP+ VEH	DFP + MPO-oral	Interaction effects P value	Statistical analysis
CA1	73.80± 4.972	110.5± 8.653	70.00± 10.22	0.3544	2-way ANOVA (Tukey's multiple comparison)
CA3	63.93± 4.224	105.0± 5.875	71.15± 9.909	0.1045	2-way ANOVA (Tukey's multiple comparison)
DG	70.17± 7.750	113.8± 5.645	70.27± 9.892	0.0521	2-way ANOVA (Tukey's multiple comparison)
AMY	84.06± 5.071	126.0± 6.064	70.97± 10.08	0.0880	2-way ANOVA (Tukey's multiple comparison)
PC	75.91± 6.903	122.5± 5.592	64.83± 6.966	0.0788	2-way ANOVA (Tukey's multiple comparison)

Figure 8B	Control	DFP+ VEH	DFP + MPO-oral	Interaction effects P value	Statistical analysis
CA1	0.1007± 0.01377	0.04144± 0.006908	0.03371± 0.005008	0.6941	2-way ANOVA (Tukey's multiple comparison)

CA3	0.08086± 0.01273	0.02800± 0.008461	0.02638± 0.005094	0.3785	2-way ANOVA (Tukey's multiple comparison)
DG	0.07462± 0.007553	0.02911± 0.009264	0.02700± 0.007901	0.7929	2-way ANOVA (Tukey's multiple comparison)
AMY	0.1202± 0.01048	0.03300± 0.008893	0.02350± 0.005301	0.7073	2-way ANOVA (Tukey's multiple comparison)
PC	0.07760± 0.009383	0.02544± 0.004901	0.02576± 0.004396	0.6537	2-way ANOVA (Tukey's multiple comparison)

Table S2. Antibodies used for IHC.

**Primary Antibody**	**Source**	**Catalogue number**	**Dilution factor**
Anti-NeuN (rabbit)	EMD Millipore	ABN78	1:200
Anti-IBA1 (goat)	Abcam	Ab5076	1:300
Anti-GFAP (mouse)	Sigma Aldrich	G3893	1:300
Anti-KIR4.1 (goat)	Santa Cruz Biotechnology	Sc-23637	1:100
Anti-GP91^{phox} (mouse)	Santa Cruz Biotechnology	sc-130543	1:100
Anti-C3 (rat)	Novus Biologicals	NB200-540	1:80
Anti-parvalbumin (rabbit)	Abcam	Ab11427	1:1000
**Secondary Antibody**	**Source**	**Catalogue number**	**Dilution factor**
AMCA blue streptavidin	Jackson ImmunoResearch	016-150-084	1:60
Cy3™-conjugated Streptavidin	Jackson ImmunoResearch	016-160-084	1:80
Biotinylated donkey anti-goat	Jackson ImmunoResearch	711-295-152	1:100
Biotinylated donkey anti-rat	Jackson ImmunoResearch	712-065-153	1:100
AlexaFluor 488 anti-mouse	Jackson ImmunoResearch	115-545-003	1:300
AlexaFluor 488 anti-rabbit	Jackson ImmunoResearch	711-545-152	1:200
Rhodamine Red X anti-rabbit	Jackson ImmunoResearch	111-295-144	1:300
Rhodamine Red X anti-mouse	Jackson ImmunoResearch	715-295-151	1:300

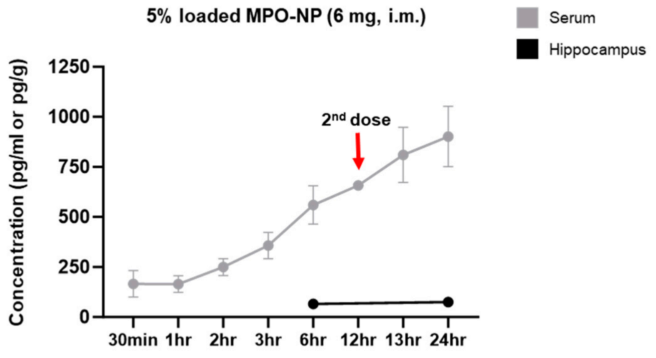

Figure S1: Serum and hippocampal MPO concentrations. In control animals given 5% loaded MPO-NP, serum (pg/mL) concentrations increased over 24 h while hippocampal (pg/g) maintained from 6 h to 24 h. n=4, data are represented as mean ± SEM.

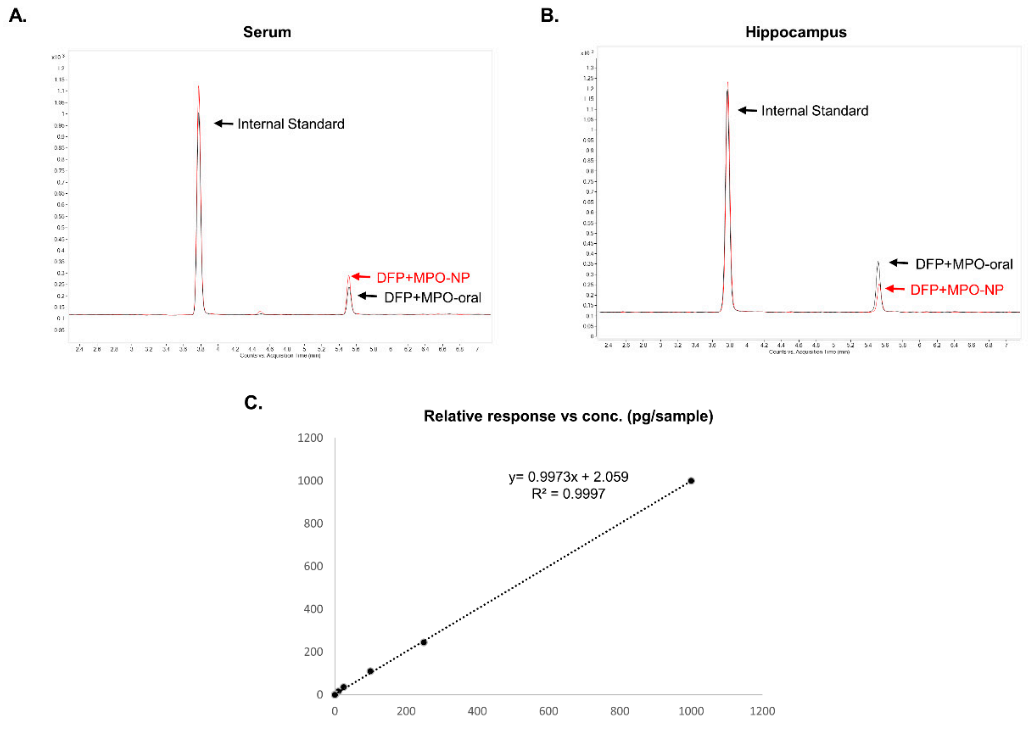

Figure S2: Method validation for MPO LCMS. Chromatograms of MPO and the internal standard in the serum (A) and hippocampus (B) on day 8. (C) The response linearity of MPO.
